# Supplementary material for: Intermittent Use of Portable NIV Increases Exercise Tolerance in COPD: A Randomised, Cross-Over Trial
Source: J Clin Med. 2019 Jan 15;8(1):94. doi: 10.3390/jcm8010094 (PMC6352193; doi:10.3390/jcm8010094)
Supplement: Supplementary file 1 [file jcm-08-00094-s001.pdf]

Table S1. Effects of the use of pNIV on anxiety, symptom burden & ability to perform tasks.

Question	Pre-VitaBreath	Post-VitaBreath	p value
How anxious are you about becoming short of breath (SOB)?<br>1= Not at all anxious<br>10 = Very anxious	7.50 (5.25-8.75)	4 (2-5.75)	0.001*<br><br>20 improvements<br>2 worse<br>2 ties
How long did it take you to recover from SOB?			0.002*<br><br>17 improvements<br>3 worse<br>4 ties
<1 min	0 (0%)	6 (25%)	
2-3 min	7 (29.2%)	9 (37.5%)	
4-5 min	3 (12.5%)	4 (16.7%)	
5-7 min	5 (20.8%)	1 (4.2%)	
7-10 min	5 (20.8%)	2 (8.3%)	
More than 10 min	4 (16.7%)	2 (8.3%)	
**Question**	**Two weeks of VitaBreath**	**Twelve weeks of VitaBreath**	
Has VitaBreath helped your ability to perform activities?			
Speed^			0.410*<br><br>6 improvements<br>4 worse<br>12 ties
1 Not at all	10 (45.5%)	8 (33.3%)	
2	1 (4.5%)	1 (4.2%)	
3	3 (13.6%)	6 (25%)	
4	6 (27.3%)	2 (8.3%)	
5 Yes, Definitely	2 (9.1%)	7 (29.2%)	
Duration^			0.856*<br><br>7 improvements<br>6 worse<br>9 ties
1 Not at all	8 (36.4%)	6 (25%)	
2	1 (4.5%)	3 (12.5%)	
3	3 (13.6%)	5 (20.8%)	
4	6 (27.3%)	3 (12.5%)	
5 Yes, Definitely	4 (18.2%)	7 (29.2%)	
Confidence§			0.972*<br><br>8 improvements<br>5 worse<br>10 ties
1 Not at all	2 (8.7%)	4 (16.7%)	
2	2 (8.7%)	1 (4.2%)	
3	7 (30.4%)	5 (20.8%)	
4	6 (26.1%)	5 (20.8%)	
5 Yes, Definitely	6 (26.1%)	9 (37.5%)	
How often do you use VitaBreath?			0.588*
Several Times a day	17 (70.8%)	15 (62.5%)	
Once a day	1 (4.2%)	1 (4.2%)	
Couple times a week	3 (12.5%)	5 (20.8%)	
Once a week	2 (8.3%)	2 (8.3%)	

Not at all	1 (4.2%)	1 (4.2%)	
What do you use VitaBreath for?~			
Waking Inside	15 (62.5%)	15 (65.2%)	n/a
Walking Outside	13 (54.2%)	15 (65.2%)	n/a
Climbing Stairs	5 (20.8%)	8 (34.8%)	n/a
Grocery shopping	2 (8.3%)	7 (30.4%)	n/a
Playing with family	2 (8.3%)	4 (17.4%)	n/a
Other physical activity	5 (20.8%)	7 (30.4%)	n/a
Indoor activities	15 (62.5%)	17 (73.9%)	0.453**
Outdoor activities	15 (62.5%)	19 (82.6%)	0.125**
How often are you breathless because of your COPD?			
Several times a day	21 (87.5%)	18 (75%)	0.672*
Once a day	0 (0%)	1 (4.2%)	3 improvements
Couple times a week	0 (0%)	3 (12.5%)	3 worse
Once a week	1 (4.2%)	2 (8.3%)	18 ties
Under once a week	2 (8.3%)	0 (0%)	
Does breathlessness stop you performing certain activities?			
Yes	19 (79.2%)	21 (87.5%)	0.50**
Do you plan activities based on the potential to become short of breath?			
Yes	20 (83.3%)	16 (66.7%)	0.219**
How would you rate VitaBreath for ease of use?			
Excellent		8 (33.3%)	
Very Good	n/a	9 (37.5%)	n/a
Good		7 (29.2%)	
Fair		0	
Poor		0	
How would you rate VitaBreath for portability?			
Excellent		3 (12.5%)	
Very Good	n/a	5 (20.8%)	n/a

Good<br>Fair<br>Poor		1 (4.2%)<br>7 (29.2%)<br>8 (33.3%)	
How active have you been since VitaBreath, compared to before?§<br><br>Median (IQR)<br><br>1(Much less active) to 4<br>5= The same<br>6 to 10 (much more active)	 <br><br><br>6(5-7)<br><br>2 (8.7%)<br>8 (34.8%)<br>13 (56.5%)	 <br><br><br>6(5-7.75)<br><br>2 (8.4%)<br>8 (33.3%)<br>14 (58.3%)	0.94*
Given your recent experience with VitaBreath, how likely is it that you would recommend VitaBreath to a friend or colleague, or other people with COPD?§<br><br>1 (Not at all likely) to 10 (Extremely likely)	Median 9 (7-10)	Median 10(7-10)	0.404*<br><br>7 less likely to recommend<br>5 more likely to recommend<br>10 Ties
Data presented as Median (IQR); Absolute number (%); *Wilcoxon Signed-rank test; ** McNemar's test; § 1 missing record at 2 weeks; ^2 missing records at 2 weeks; ~ 1 record missing at 12 weeks.			
